# Parenting on the margins: a qualitative study of the challenges encountered by fathers of children with autism in Limpopo Province, South Africa

**DOI:** 10.3389/fpsyt.2025.1730241

**Published:** 2026-01-05

**Authors:** M. Rosinah Nkgaphela, G. Olivia Sumbane, L. Sebolaisi Hlahla, L. Winter Mokhwelepa

**Affiliations:** 1 Faculty of Health Science, School of Health Care Science, Nursing Science Department, University of Limpopo, Polokwane, South Africa; 2 Faculty of Health Science, School of Medicine, University of Limpopo, Polokwane, South Africa

**Keywords:** challenges, fathers, children, autism, ASD

## Abstract

**Background:**

Autism spectrum disorder (ASD) has a profound impact on families, including fathers, who often face multiple challenges while raising their children. Fathers of children with ASD frequently experience stress, financial difficulties, stigma, and limited support, which can negatively affect their wellbeing and caregiving capacity.

**Methods:**

A qualitative, phenomenological research design was employed to gain a detailed understanding of the challenges faced by fathers. Non-probability purposive sampling was used to recruit 15 fathers of children with ASD. Data were collected through semi-structured interviews and analyzed using Tesch’s eight steps of data analysis.

**Results:**

The findings showed challenges directly related to ASD, social challenges associated with caring for children with ASD, and service provision challenges experienced by fathers.

**Conclusion:**

Fathers of children with ASD face a wide range of difficulties that extend beyond caregiving and include difficulties in the management of behavioral problems, inadequate service support, and societal stigma. An effective intervention program should incorporate the unique challenges and experiences of fathers of children with ASD to ensure that they receive appropriate assistance. These findings can be used to monitor and evaluate the support provided to fathers of children with ASD.

## Introduction

Fathers play a vital but historically poorly understood role in raising children with autism spectrum disorder (ASD). Research worldwide shows that the prevalence of ASD is increasing, and raising a child with ASD can produce prolonged demands on families. However, the majority of studies on caregivers centralize mothers, neglecting the fact that fathers’ experiences are not as respected in the literature (1, 2). This gap matters because fathers contribute uniquely to a child’s development (e.g., style of play, discipline, and socialization) and family resilience. Interventions that do not include fathers miss opportunities to change the outcomes for the entire family (2).

Fathers of children with ASD describe a complicated mixture of grief, uncertainty, anxiety, and, often, symptoms of depression. In qualitatively focused studies, fathers described their shock at their child’s diagnosis, recurring worries about their child being independent in life, and their feelings of failure or guilt that arise when their child exhibits challenging behavior in public (3, 4). For example, fathers recounted sudden confrontations at school meetings characterized by their child’s behavior that led to threats of exclusion, which later led to anticipatory anxiety and sleepless nights about the options available once their child needed housing or employment. Quantitative studies have also shown elevated rates of caregiver distress and depressive symptoms among the caregiver populations involved in the care of children with autism, with some studies indicating that fathers experience greater mental health burdens than mothers, although patterns may be rarely comparable (5, 6).

The financial and practical burdens that fathers may experience can be severe and complex. Fathers report, at times, higher out-of-pocket expenses (e.g., therapy, specialized schools, and travel), lost hours of work, and potentially even the loss of higher paying jobs when they adopt a parent or caregiver or advocacy approach (7, 8). As illustrations, fathers report giving up full-time work for part-time work due to the variety of therapy appointments, paying for private speech or behavioral therapy when it is not available adequately in a public program, and making commitments to ongoing travel costs for specialist appointments, including multispecialty appointments (9). The financial implications do elevate the stress and can disrupt a family’s financial plans [e.g., having (more) children and mortgage decisions].

In Sub-Saharan and in many low-resource contexts, social isolation, stigma, and cultural responses create various barriers. Studies from Kenya and East Africa and from a broader Sub-Saharan perspective have illustrated that fathers experience community stigma (i.e., blame and rumors), that autism awareness is limited, and that families often prefer spiritual or traditional practices, further delaying diagnosis and the associated appropriate intervention (10, 11). In this context, fathers may find pressure from extended family members to hide the child or seek faith-healing rather than therapy, and those who push back against pressure risk being considered a “weak” father or being alienated from the community, exposing the family to further social isolation (10).

However, the limited research from South Africa demonstrates how the interaction of service gaps, cultural constructions of fatherhood, and health inequities shapes the experiences of fathers in service settings. South African qualitative studies, both phenomenological research and exploratory interviews, found that fathers described the renegotiation of their role (becoming primary advocates, navigating schools, and navigating doctors), the emotional labor (masking distress to maintain family functioning), and the frustration with long waits for an ASD diagnosis or the lack of local therapists trained in ASD (12, 13). South African fathers reported being re-referred between clinics, paying for private assessments, or being disregarded during clinical consultations because it was assumed that fathers are not the primary caregivers for children with disabilities. These findings are consistent between jurisdictions (i.e., lack of knowledge and limited community resources) and create a greater need for policies that include fathers in South African mental health and education policy (12). Therefore, this study aimed to explore the challenges faced by fathers of children with ASDs at a special school selected in Limpopo Province.

## Theoretical framework

**Figure 1:**
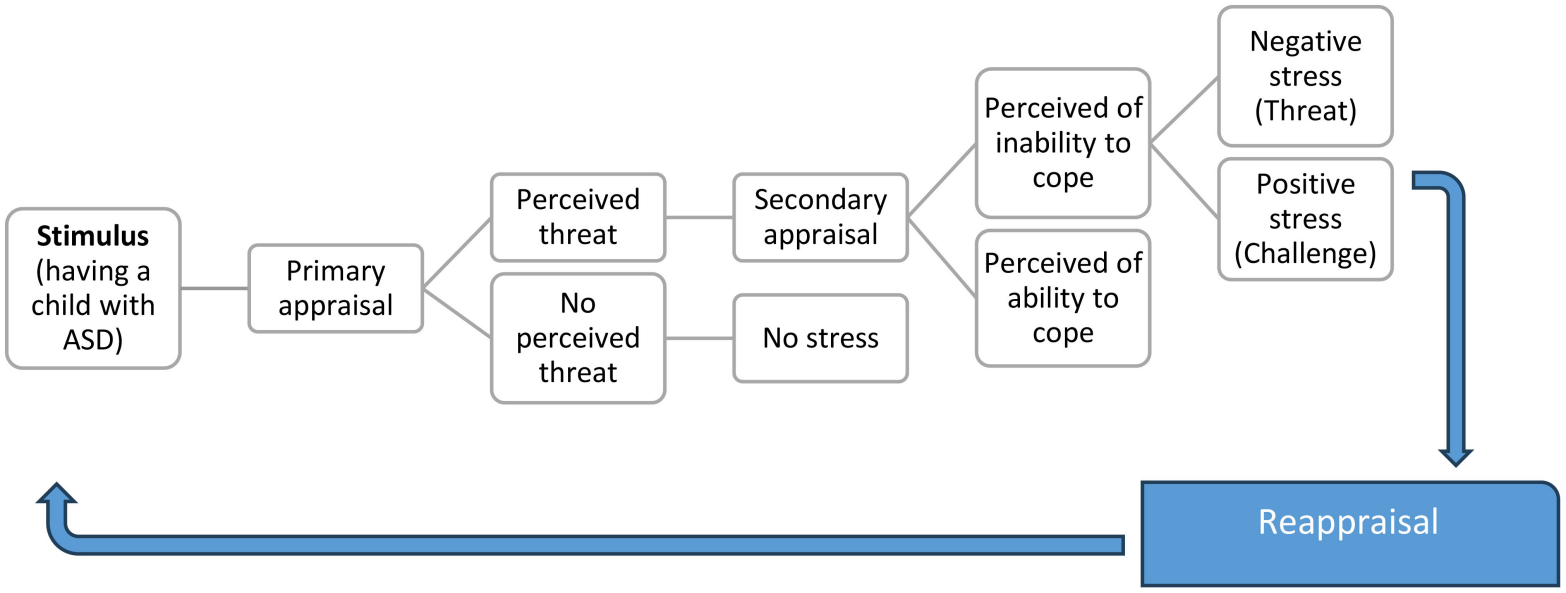
Lazarus and Folkman’s 1984 transactional model of stress and coping (14).

The study was guided by Lazarus and Folkman’s 1984 transactional model of stress and coping (14). The theory claims that stress stimuli are most commonly thought of as events that impinge on the person. Stimulus definitions also include conditions that arise within the person. An event itself is not a stressor. Instead, the event is considered a stressor only after a person has assessed it as harmful or threatening within the limits of the environment. According to Lazarus and Folkman, there are three types of appraisals: primary, secondary, and reappraisal (14). In the primary assessment, a person determines whether an event or situation poses a threat or challenge. If the demands are greater than the available resources, the individual may perceive the situation as threatening or harmful. Therefore, the event is a stressor. If the resources at hand are greater than the demands, then the situation may be considered a challenge and potentially beneficial. Therefore, the event is not a stressor. This is illustrated in **Figure 1**.

If the situation is deemed a threat, then a secondary assessment kicks in. During the secondary evaluation, people evaluate their own ability to deal with the stressor and analyze which coping mechanisms would be the most effective. Coping techniques include avoiding, diminishing, changing, or accepting a stressful situation.

Reappraisal involves repeatedly evaluating and modifying the perceptions made during the primary appraisal or the secondary appraisal. In an ongoing situation, the perceived threat may later be construed as a challenge. Over time, the stressor may be considered harmless or irrelevant. Alternatively, challenges can become stressful and become threats over time.

In this study, having a child with ASD was considered as the stress stimulus. It depends on the father of the child with ASD whether the child with ASD poses a threat or a challenge. If the resources needed for raising a child with ASD are greater than the available resources, the father may perceive the situation as threatening or harmful. Therefore, having a child with ASD will be a stressor. If the resources available for raising a child with ASD are greater than the demands, then having a child with ASD can be considered a challenge and potentially beneficial.

## Materials and methods

### Setting

The research was conducted at a selected special school in Limpopo Province. This special school stands as the only establishment within Limpopo Province that accommodates the highest population of learners diagnosed with ASD and comprises three classrooms dedicated exclusively to learners with ASD. Autism-specific classes are stratified according to educational phases, specifically the junior, middle, and senior phases. The total number of ASD learners in each classroom ranges from 12 to 13. The selected institution enrolls learners with ASD from across the entire Limpopo Province. The educational methodology employed in these institutions primarily focuses on developing vocational skills.

### Research design

A qualitative and phenomenological research design was used to obtain a detailed description and understanding of the challenges faced by fathers of children with ASD.

### Sampling of participants

The total number of ASD learners with fathers in the selected school was 26. A total of 15 fathers of children with ASD were selected using a non-probability purposive sampling technique. Of these, 14 were the biological fathers of children with ASD, while one father was a foster parent of a child with ASD. Their ages ranged from 34 to 63 years. Of the fathers, 10 were married, two were single, one was widowed, one was engaged, and one was divorced. **Table 1** summarizes the characteristics of the participants.

**Table 1:** Characteristics of the participants.

Participants	Age (years)	Marital status	Highest qualification	Relationship with the child	Age of the ASD child (years)	Gender of the child
P1	63	Widow	Standard 1	Foster father	14	Female
P2	35	Married	Degree	Biological father	6	Male
P3	46	Married	Diploma	Biological father	7	Male
P4	41	Single	Grade 12	Biological father	15	Male
P5	41	Married	Diploma	Biological father	8	Male
P6	40	Engaged	Grade 12	Biological father	5	Male
P7	55	Married	Degree	Biological father	12	Female
P8	53	Married	Degree	Biological father	14	Male
P9	41	Divorcee	Degree	Biological father	9	Male
P10	47	Married	Grade 12	Biological father	7	Male
P11	40	Married	Grade 12	Biological father	9	Male
P12	43	Married	Grade 12	Biological father	8	Female
P13	45	Married	Grade 12	Biological father	12	Male
P14	45	Married	Grade 12	Biological father	09	Male
P15	34	Single	Grade 12	Biological father	08	Male

### Data collection

Data were collected through semi-structured interviews conducted by the researchers. A total of 15 interviews were conducted, comprising 12 face-to-face and three telephone interviews. The telephone interviews were conducted for fathers who reside far away and did not wish to travel to the school for the interviews.

There were 12 face-to-face interviews conducted in the classrooms after school to ensure privacy, facilitate clear audio-recording, and minimize interruptions. Similarly to the telephone interviews, the researchers ensured the location was noise-free and that it was situated in a quiet, private room. The participants were also asked to sit quietly in a private room so that only the researcher and the participants could hear what was said during the interview. Thus, to prevent interruptions from incoming calls during the interview, the researcher pre-programmed her mobile phone to divert any calls to her mother’s cell phone before phoning the participants. The interview expectations, including the types of questions to be asked, the duration, the desired name to be used for anonymity, and the usage of a tape recorder, were first explained to all participants by the researcher.

The interviews were audio-recorded, and field notes were taken to capture emotions and nonverbal cues. Majority of the participants preferred to be interviewed in Sepedi, and the central question guiding the interviews was: “Kindly describe the challenges that you faced as a father of a child with ASD.” A brief interview guide with open-ended questions and probing prompts supported the discussions. Each interview lasted 30–45 min and was conducted between February and April 2024. Data collection continued until saturation was reached at the 10th interview, with five additional interviews conducted to confirm saturation. Throughout the interviews, the researcher used techniques such as probing, summarizing, and paraphrasing to facilitate rich, meaningful responses.

### Ethical considerations

Ethical clearance to conduct this study was obtained from the University of Limpopo’s Turfloop Research Ethics Committee (TREC/1691/2023). The Department of Basic Education and the principal of the selected special school granted permission to conduct a study at the school.

The researcher first visited the chosen special school to inform the principal about the study’s goals and scope and to request permission to conduct the research. Approval was granted to conduct the research at the special school. The researcher was notified by the principal that the parents of children with ASD would be attending an ASD awareness event at the school. Therefore, the researcher was invited to attend the awareness event in order to facilitate the recruitment of fathers of children with ASD. Following the autism awareness event the researcher attended at the special school, the majority of the fathers were recruited. Each person who consented to participate in the study signed a consent form. All those who gave their consent were scheduled for data collection at a convenient time and location.

### Data analysis

Data were analyzed using a thematic approach (15). The first step involved transcribing the interviews verbatim to organize the data. As the interviews were conducted in Sepedi, they were first transcribed in Sepedi and then translated into English. Both the transcription and translation were carried out by a trained bilingual research assistant fluent in Sepedi and English, and the translations were later checked by the researchers for accuracy. To gain a comprehensive understanding of the material and consider its overall significance, the researchers carefully reviewed all the data, including the transcriptions and field notes. As they studied the data, they asked themselves reflective questions such as, “What is this participant saying?” and noted every idea that came to mind. A brief and engaging interview transcript was selected and read through, with emerging thoughts recorded in the margins. To develop an initial summary of the data, the researchers began with the shortest and most compelling transcripts. Throughout the process, they continued to ask, “What is this about?” in order to uncover the underlying meaning within the data.

A list of every topic was created after reviewing the transcriptions, grouping together related topics, and organizing these topics into columns designated as main, unique, or leftover topics. After reviewing the data, the list of topics was used to shorten the themes into codes, which were then written next to the relevant textual passages. The researchers did this to determine whether any new codes or categories had been identified. To limit the number of categories, related topics were grouped together, and then lines were drawn between the categories to illustrate their interrelationships. The codes were arranged alphabetically, and the final abbreviation was determined for each group. Data from each category were compiled, and an initial analysis of the data was carried out. Existing data were inspected according to themes and sub-themes.

To validate the data, all researchers listened to the voice recording and discussed the emerging themes. An independent coder provided the transcripts. The final themes and sub-themes were decided upon during a meeting between the researchers and the independent coder.

## Results

Three themes and nine sub-themes emerged from the findings, as illustrated in **Table 2**. The study revealed that fathers of children with ASD face multiple caregiving challenges, including challenges related to ASD, social challenges related to caring for children with ASD, service provision challenges for children with ASD, and psychological challenges. The themes and sub-themes that emerged are illustrated in **Table 2**.

**Table 2:** Study findings.

Themes	Sub-themes
1. Challenges related to ASD	• Behavioral disturbances and impaired emotional regulation and impulse control • Physical and psychomotor disturbances • Communication challenges
2. Social challenges related to the care of children with ASD	• Inadequate social awareness of ASD • Social rejection and stigma • Family burden • Financial burden
3. Service provision challenges for children with ASD	• Challenges in finding a suitable educational facility • Inadequate healthcare support

*ASD*, autism spectrum disorder.

### Theme 1: challenges related to ASD

The difficulties faced by fathers of children with ASD were the basis of this theme. According to this study, the fathers of children with ASD face several difficulties in raising their children. The challenges faced by fathers of children with ASD were as follows: behavioral disturbances, impaired emotional regulation and impulse control, physical and psychomotor disturbances, and communication challenges.

#### Behavioral disturbances and impaired emotional regulation and impulse control

As the majority of children with ASD have behavioral issues and behave inappropriately, it was established by this study that the fathers of children with ASD have certain challenges as a result of their child’s ASD behaviors. Data suggest that the behaviors children with ASD have include being aggressive by threatening family members with a knife, beating others, smashing windows, fighting themselves to the ground, hitting the cupboard with their head, stealing food from neighbors, and having frequent temper tantrums. It was stated that fathers find it difficult to manage this type of activity. As evidenced by:

*“Eiii, this one-off is very angry when he does not get what he wants, eiii, he gets angry with that boy. I am always afraid that he will get injured because he throws himself to the floor when he is angry. He once hit the cupboard with his head back then because he was angry. We try to manage this kind of behavior, but it’s very difficult to deal with it. It’s difficult because if he can get angry while there is no one next to him, he will start throwing himself on the floor and end up getting hurt.”* (P10)

*“I do not know what can be done to help us so that what we are experiencing will be better and not be the way it is now. Now that schools are closing, I don’t know what to do because he is coming back; even his siblings will say so. He only hits his siblings; sometimes he hits them and ends up taking a knife and threatening to stab them. He is seven years old, but has excessive power.”* (P3)

#### Physical and psychomotor disturbances

The physical and psychomotor disturbances of their children with ASD were mentioned by the participants as a challenge. According to some of the fathers, their children are hyperactive, never sleep at night, run around the yard all the time, have fussy eating habits, and sometimes eat a lot. Due to their children’s constant wandering, hyperactivity, and inability to sleep at night, some of the fathers of children with ASD are always on guard. Because their children with ASD overeat, some parents need to purchase additional food for the household. These behaviors negatively impact some marriages and are inconvenient for fathers of children with ASD. As evidenced by:

*“That’s why I am saying this is of concern because he does not sleep. When he is around, he doesn’t sleep, and he wants to sleep with his mother; he can even spend the whole day and night without sleeping and sometimes sleeps at 2 am. It is distressful and affects our marriage.”* (P3, 46-year-old father)

*“We do not lock it because he does not go to the gate when he is around. He just runs around the yard and the house. He does not watch television at all.”* (P6, 40-year-old father)

*“He does not eat some things; he does not eat cheese or milk. Everything that has milk in it is not eaten.”* (P4, 41-year-old father)

#### Communication challenges

Fathers were also found to face communication difficulties with their children with ASD, as the majority of them have difficulty speaking and some of them cannot speak at all. Due to the speech problems of their children, the fathers often find it challenging to interact with them. They said that they occasionally miss what their children are saying and instead assume what they might desire. Because children with ASD are unable to express their demands, the majority of the fathers appear to be concerned about their lack of communication skills. As evidenced by:

*“Speech is also a problem; He talks, but not well. Sometimes I understand, and sometimes I don’t, so I have to try to understand him. This one-off being unable to talk well gives me a problem.”* (P9)

*“Having an ASD child is very difficult, especially when he is not able to talk. You have to understand what this child wants because he cannot talk.”* (P3, 46-year-old father)

### Theme 2: social challenges related to caring for children with ASD

The results of this study revealed that fathers experience the following social challenges related to caring for children with ASD: inadequate social awareness of ASD and inadequate healthcare support, social rejection and stigma, family burden, and financial burden.

#### Inadequate social awareness

The study demonstrated that the majority of family members and members of the community are unaware of ASD. According to fathers, a lack of social awareness about ASD in the community causes community members to be ignorant of ASD. Some members of society believe that parents who indulge their children with ASD are to blame for the behavior of these children. Some fathers stated that, after having a child with ASD, they became aware of the disorder. The community views children with ASD in a certain way due to a lack of awareness. As evidenced by:

*“When we are in the mall, he runs around the mall, so I realized that most people do not know that there is a condition called autism because they will just stare at him, thinking that maybe he is just troublesome and doesn’t hear when rebuked or something like that. They just ask themselves, why are we not holding our child? When we hold him, he becomes irritated and screams, and when you stop holding him, he runs around the mall.”* (P6)

*“The problem is the people in the community. When I am with my son and meet people on the street, and my son starts to behave in some way, I have to explain to those people the reason for my son’s behavior and start explaining what ASD is, and I find that it takes more time for him to understand what ASD is.”* (P8, 53-year-old father)

#### Social rejection and stigma

The outcomes of the study revealed that fathers of children with ASD face social rejection and stigma from the community. They are being discriminated against and are not treated well by the community because they have children with ASD. Their children with ASD are also not treated well by the community. This could be due to a lack of knowledge about ASD in the community. Some parents do not allow their children to play with children with ASD; some even make jokes about children with ASD and call them names. This kind of behavior seems to bother the majority of fathers as some of them reported that they are no longer getting along with their neighbors, and some are stressed by this. As evidenced by:

*“‘The society views my child as stupid; they don’t interact well with him.”* (P2)

*“The problem is our neighbors and their children; they don’t want him. They do not want to play with him. Sometimes he even cries, wanting to play with them. Sometimes, when he plays with other children, he does not beat them. He does not beat others; they just do not want to play with him and do not accept him in the community. Even mothers, when they see him, chase him out of their yard and lock their gates. They do not want him; they just say it in public.”* (P3)

#### Family burden

This sub-theme developed from the fathers’ explanations of how having a child with ASD strained the family. According to some fathers, having a child with ASD negatively impacts the marriage. Some marriages are different now than before having a child with ASD, while other parents are almost divorced. They added that having a child with ASD leads to disputes and clashes between them and their spouses. Children with ASD are the ones who receive all the attention. Parents no longer have time for themselves, and siblings do not get much attention from their parents. As evidenced by:

*“It affected our marriage too much. We almost divorced. Having a child with ASD led to numerous conflicts. It sparked numerous arguments; we asked ourselves who had brought it and what had happened. We argued a lot and blamed each other; I said it came with her, and she also said no, it came with me.”* (P7)

*“My wife and I are always fighting; we always have disagreements because I would say, ‘Let’s do this and his mother would say, No, let’s do that.’ Eishhh, it is tough. His mother no longer has time for me; she is always focusing on this child. It is hard because we share a room with him, and he is old. We are no longer able to fulfil the roles of a wife and husband as they are traditionally defined. My wife ended up accusing me of cheating.”* (P10)

#### Financial burden

The data provided details on the financial burden on the fathers of children with ASD. Seven fathers were unemployed, two were pensioners, and six were employed. It has been reported that financial constraints are a challenge when raising children with autism as the needs of these children are too expensive. All of the fathers indicated that the medical expenses, the need for a specialized doctor, the purchase of clothing, food, school uniforms, toiletries, and school transportation, as well as other expenses, are very expensive for them to pay. The fathers saw this as a threat because they could not meet all the needs of their children. As evidenced by:

*“The issue of money becomes a problem because now we are approaching Good Friday. Good Friday clothes are needed. She will also have to go back to school, and money for school fees and toiletries is needed. Sometimes I have to use my social grant money, which is intended for food, and combine it with yours so that I can pay her school fees, buy her toiletries, and buy her clothes because your social grant does not cover all her needs. This does not sit well with me. During the school holidays, I also have to buy enough food for her to eat until she goes back to school.”* (P1)

*“The child has to go see an occupational therapist and a speech therapist every month. They also sometimes refer the child to a neurosurgeon, and when you arrive there, they want co-payments because this is the first visit. Most neurologists need R2700, and the child grant is R2000. We have not yet counted the child’s needs.”* (P2)

### Theme 3: service provision challenges

This theme was based on the challenges of the health and educational services for children with ASD in Limpopo Province as experienced by fathers.

#### Challenges in finding a suitable educational facility

Limpopo Province is South Africa’s most rural area and has no school for those with autism. However, there are only two schools that offer classes for autistic learners. Consequently, the fathers explained that it was extremely difficult to enroll their children with autism as there were many students on the waiting list and only a few classes for those with autism. Majority of the fathers recognize that they have to fight, walk up and down, and present the necessary documents. Some of them said that, despite repeated claims by schools that their children did not have space, they were still accepted the following year.

As evidenced by:

*“So, as he grew up, I went to a special school to look for a space for him, and they said I had to bring home letters from the hospital, so I went to the hospital to get those papers. When I returned with those papers, they said there was no space and that we must wait until next year. It was difficult to get the space.”* (P10, 47-year-old father)

*“It was difficult. I struggled for almost 2 years. There was no room for him at the special school. They also wanted several things before admitting him to the special school, but finally they ended up taking him.”* (P11)

#### Inadequate healthcare support

Another challenge relates to inadequate health support. Some fathers are dissatisfied with the services they receive from healthcare facilities. The fathers emphasized that the treatment of their children with autism does not improve their condition or their behavioral problems. Furthermore, the fathers reported that they did not receive sufficient information on the condition of their children. This leads to difficulties in managing their children’s behavioral problems. This is supported by the following quotes:

*“My child started talking when he was 3 years old. I was not satisfied with the service the hospital provided to the child; they just said go home and when the child is not well, you must bring him back.”* (P2)

*“He is taking medication, but I think the pills he is taking are making him worse. They do not even work for him. They just said that the medication is okay as it is and that it will improve when he grows up.”* (P3)

## Discussion

The researchers explored and described the challenges faced by the fathers of children with ASD in Limpopo Province, South Africa. According to the study, the fathers of children with ASD perceived having ASD children as a challenge or a threat. Their perceptions were dependent on the circumstances faced while raising a child with ASD. Fathers faced challenges in managing behavioral problems such as hyperactivity, aggression, tantrums, self-harm, impaired communication, and eating habits. They also struggled with their children’s sleeplessness at night. The literature shows that the challenges of the parents of children with ASD are influenced by the child’s behavioral problems and deficits in socialization and communication (16–19).

Another situation that threatens and challenges fathers is the way in which the community treats their children with autism. This happens because community members are not aware of what autism is. It was reported that children with ASD are stigmatized, rejected, called different names, and even joked about. According to Boshoff et al. (20), society lacks knowledge, awareness, or concern for children with autism, which leads people to interpret these children’s behavior as poor parenting. Alareeki et al. (21) stated that the majority of children with autism are rejected by society because of the lack of awareness of autism.

Having children with ASD has an impact on the entire family because parents tend to pay more attention to the child with ASD than to other family members. This situation has also been reported to be a challenge for fathers because mothers spend most of the time caring for their children with ASD, both day and night. As a result, they have less time to spend with their partners. Their marriages suffered and were threatened because the fathers were accused of cheating. Other partners felt that they were not given enough attention. This ultimately led to conflicts and arguments at home. Similarly, previous research has shown that having children with autism has an impact on most marriages because parents spend all their time and energy raising children rather than raising themselves (18). The burden of raising children with autism causes conflicts among ASD parents (22, 23).

Another challenge for fathers was that the needs of children with ASD are higher than the available budget. Due to financial constraints, the fathers discovered that they could not cover essential expenses for their children with autism. These expenses include medical care, clothing, food, school uniforms, toiletries, and school transport. The cost of raising children with autism is estimated to be higher, including visits to a specialist, educational costs, medication purchases, and special diets (18, 22, 24).

Another challenge was that fathers felt ignored and neglected by health professionals due to them not having enough information about ASD. Furthermore, finding an appropriate school for children with autism presents a serious challenge in Limpopo Province. This is because there are only two special schools with a limited number of classes that specifically accept learners with autism. The findings are similar to those reported in the study by Sumbane et al. (25).

According to this study, fathers of children with autism in Limpopo Province face various challenges while raising their children with autism. They lack sufficient support to meet the needs of their children. Consequently, the situation poses a threat to these fathers because the demands of autistic children are greater than the available resources. These can have a negative impact on the father’s wellbeing and caregiving capacity. However, the South African National Mental Health Policy Framework 2013–2020 emphasized that families with intellectual disabilities, including those with ASD, must receive the maximum support to expand the support and care network (26). It is therefore necessary to provide integrated services and social support for fathers of children with ASD. The findings were merged with Lazarus’s and Folkman’s 1984 stress and coping theory, as fathers perceived raising children with autism as a challenge (14, 27). The burden of caring for a child with ASD increased due to difficulties in managing their behavioral problems. Adding to these challenges are issues of community rejection and stigmatization faced by the children and their families. Furthermore, the needs or demands of a child with ASD are greater than the available resources.

The study recommends integrating the challenges and experiences of the fathers of children with autism in order to develop an intervention program that can be used as a guide to support fathers of children with autism. The fathers of children with ASD should receive comprehensive information about the condition immediately after diagnosis. This will help ensure that they are well informed and prepared for the future. To promote awareness of ASD in communities, schools and health institutions should provide accurate information to the public. This will improve people’s knowledge about the diagnosis of ASD and reduce the stigmatization of children with this condition.

## Conclusions

This study brings to light the challenges faced by fathers raising children with ASD in Limpopo Province. The findings showed that fathers feel that raising children with autism is a challenge because it is difficult to cope with the behavioral problems of children with autism, the community rejects and stigmatize children with autism, there is a lack of support services, and because of the family and financial burden of raising children with autism. These conclusions can be used to monitor and evaluate support for children with autism in rural areas of Limpopo Province. Furthermore, these findings can inform the development of family support interventions for fathers raising autistic children in rural South Africa.

## Data Availability

The raw data supporting the conclusions of this article will be made available by the authors, without undue reservation.
